# Soft Tissue Sarcomas: Is Pre-operative Radiotherapy Associated With More Acute Wound Complications?

**DOI:** 10.7759/cureus.15654

**Published:** 2021-06-15

**Authors:** Nicholas J Rene, Alejandro Castiglioni, Nicolás Cóccaro, Bárbara Scheitlin, Lucía Papa

**Affiliations:** 1 Radiation Oncology, Centro de Radioterapia, Rosario, ARG; 2 Orthopaedic Oncology, Sanatorio Britanico, Rosario, ARG; 3 Radiology, Sanatorio Britanico, Rosario, ARG; 4 Oncology, Sanatorio Britanico, Rosario, ARG; 5 Biostatistics, Sanatorio Britanico, Rosario, ARG

**Keywords:** external beam radiation therapy, soft tissue sarcoma, wound complication, local treatment, delayed treatment, suspended treatment

## Abstract

Introduction: Increased wound complication rates are attributed to the use of pre-operative radiotherapy. The purpose of this study is to evaluate the incidence of complications with or without pre-operative radiotherapy in our institution.

Methods: We retrospectively evaluated 48 adult patients with high-grade extremity soft tissue sarcoma. Twenty-two patients received pre-operative radiotherapy (group A) while 26 patients underwent initial surgery (group B). Complications were defined as major wound complications if they were severe enough to delay the delivery of adjuvant treatment (chemotherapy or radiotherapy) more than eight weeks after surgery or if their resolution required a new surgical intervention.

Results: Mean follow-up in group A and group B was 44.3 and 53.8 months, respectively. The incidence of complications of any grade in group A was 45.5% and 53.8% in group B (p: 0.566). Major wound complications in group A and group B occurred in 18% and 23% of the patients (p=0.630), respectively. All patients in group A completed local treatment - radiotherapy and surgery - in 66 days on average. In contrast, in group B post-operative radiotherapy was either delayed or suspended in four patients due to wound complications. This determined that 15.4% of the patients in group B did not receive the local treatment - surgery + radiotherapy - as planned.

Conclusions: An increased risk of severe acute wound complications with the administration of pre-operative radiotherapy was not observed in patients with soft tissue sarcomas of the extremities. In addition, local treatment completion was not jeopardized with preoperative radiotherapy, as opposed to post-operative radiotherapy.

## Introduction

Soft tissue sarcomas (STS) comprise a heterogeneous group of infrequent tumors with a diverse age and site of presentation, different evolution and prognosis. As a group, STS represent approximately 1% of all cancers in adults [1]. 

Like in many areas of oncology, the treatment of high-grade STS has evolved from radical surgical procedures (i.e., amputations) to more conservative surgeries, currently consisting of tumoral resection with margins. Surgery remains the mainstay of local treatment. Radiation therapy (RT) is usually associated with surgery to achieve better local control rates in cases of high-grade histology, deep location, inadequate surgical margins or in recurrent cases [2-4]. Randomized trials established that the combination of a conservative surgery plus RT is safe and yields a good quality of life [5,6]. Five-year survival rates of 53% are currently achieved in patients with high-grade STS [7].

Nowadays, the debate on the local treatment of STS is the timing at which RT should be delivered. Pre-operative RT may be associated with more acute wound complications. In contrast, higher radiation doses used in post-operative RT can result in more chronic, irreversible complications [8,9].

In this study, we report our institutional experience of patients treated with limb-sparing surgery for STS. The rate of acute post-operative wound complications is analyzed according to whether or not pre-operative radiotherapy was administered. In patients treated with surgery plus RT, the rate of completion of the local treatment is also analyzed depending on the time at which radiotherapy was delivered.

## Materials and methods

In 2010, a multidisciplinary team dedicated to the treatment of sarcomas was started at our institution and all patient´s data were prospectively accrued.

Between August 2010 and December 2016, 96 patients with extremity non-metastatic STS were operated on. Forty-eight patients were excluded from this analysis because of the following reasons: Younger than 18 years of age; low-grade histology; superficial location; amputations; tumor in a previously irradiated area; tumoral cutaneous complications (tumoral ulcers).

Patients were divided into two groups depending on whether or not RT was administered before surgery. Group A includes 22 patients who were first treated with pre-operative RT and then operated on. Meanwhile, Group B consists of 26 patients who underwent initial surgery. In the beginning, for patients in whom RT was considered necessary, a post-operative RT regimen was used. Later, in view of possible benefits, our treatment policy changed to pre-operative RT [7,10,11].

Wound dehiscence, infection, necrosis and seroma of any grade were considered complications and were recorded. We defined as major complications those that were severe enough to delay the delivery of adjuvant treatment (chemotherapy or radiotherapy) more than eight weeks after surgery or if its resolution required a new surgical intervention.

Follow-up time was calculated from the first local treatment that each patient received (from the start of RT for patients receiving pre-operative RT or from the date of surgery for patients that were initially operated on).

Diagnostic methods and biopsy

Local tumor extension was assessed with MRI in all the cases. In addition, MRI was used to define the site of biopsy in areas of contrast enhancement or areas of restriction in the diffusion sequence. In order to evaluate any changes induced by the treatment, patients in group A underwent MRI scans before the start, and after finishing, the course of radiotherapy. All the patients were studied with a contrast-enhanced CT scan of the chest, abdomen and pelvis. Patients with leiomyosarcoma also performed a bone scan.

Radiation therapy

Patients were treated with a 3D conformal technique with six-MV photons. Special attention was paid to avoid areas of high doses of radiation (“hot spots”). Group A patients received a total dose of 50 grays (Gy) in 25 daily fractions of 2 Gy each, five days a week. The typical volume included the tumor with a 4-5-cm margin in the longitudinal direction and 1.5-2 cm radially. Edema was also included with a 1-2-cm margin. Margins were adjusted in areas of natural barriers like bone or fascia. When RT was administered after the surgery (group B) the first phase of 50 Gy in daily 2 Gy fractions was administered to the surgical bed, including all the scars and drainage sites. Treatment margins were similar to group A patients. The second phase of 10-16 Gy was administered to the tumor bed with 2 cm margins. Bolus (tissue-equivalent material) was used in all cases over the scars and drainage sites to avoid under-dosage of these areas.

Radiotherapy was indicated in all patients with deep tumors, superficial tumors larger than 5 cm, cases in which close surgical margins were obtained (post-operative RT) or anticipated (pre-operative RT) and in recurrent tumors, but the final decision was defined in a case by case basis. As mentioned earlier, in the beginning, RT was mainly administered post-operatively and then a pre-operative regimen was preferred.

Surgical procedure

Surgery consisted of the resection of the whole lesion with normal tissue margins around it. Resection included all the previous scars and drainage sites. Special attention was paid to minimize the resection of normal tissues. In some cases, an ultrasound scan was performed to define the best way to access the tumor without resecting unnecessary normal tissues. In order to reduce the probability of deep seroma formation, hemostasia was meticulously maintained throughout the procedure, aspirative drainage catheters were always left in place for two to three days and elastic bandages were used. All reconstructions were made with primary closure. Rehabilitation was started only after the wound was in adequate condition.

The resected specimen was evaluated in-fresh in the pathology laboratory. A negative margin was obtained in all cases, except in one patient with a large tumor in the shoulder who had a positive margin in the rib cage. Immunohistochemistry methods were used when necessary to define histologic subtype.

Radiotherapy and surgery were performed by the same teams in all the cases. In group A, surgery was performed between three and eight weeks after the end of RT.

Post-operative care and follow-up

After surgery, patients were evaluated weekly until the wound was completely healed. Afterwards, they were seen every three months for two years and every six months thereafter. An MRI scan of the surgical bed and a CT scan of the thorax, abdomen and pelvis were performed every six months.

Statistical analysis

Chi-square homogeneity test for categorical variables and U Mann-Whitney test for quantitative variables were used to determine if the two groups (Group A and Group B) belonged to the same population and were comparable. Chi-square homogeneity test was used to compare the evolution and acute complication rate of the wounds in both groups of patients. Also, it was used to compare the local recurrence rate among groups. A log-rank test was used to compare the survival rate of the groups. All statistical analyses were performed using the R software (version 3.6). Differences were considered statistically significant at a p‐value < 0.05.

## Results

Patient, tumor and treatment characteristics

Patient characteristics were compared between group A (n: 22) and group B (n: 26). Both groups resulted to be statistically homogeneous, comparable and belonging to the same population (Table 1).

**Table 1:** Clinicopathologic characteristics of the 48 evaluable patients with soft tissue sarcoma AJCC: American Joint Committee on Cancer Staging

Variable	Group A (n:22)	Group B (n:26)	P-value
Sex	15 females : 7 males	11 females : 15 males	0.07
Mean age (years)	48.27 (SD = 17.8)	52.88 (SD = 18.67)	0.438
Delay in diagnosis (months)	8.73 (SD = 6.86)	8.03 (SD = 6.56)	0.883
Site	Lower Limb: 70.8% (Thigh 60.4%) Upper Limb: 29.2%	Lower limb: 72.7% (Thigh 63.6%) Upper limb: 27.3%	0.270
Mean size (cm)	12.75 (min: 7.1/max: 27.5)	11.87 (min: 4.5/max: 29.1)	0.414
Enneking stage	IIA (54.5%)/IIB (45.5%)	IIA (53.9%)/IIB (46.1%)	0.21
AJCC stage (8th Ed.)	IIB (4.5%)/IIC (4.5%)/III (91%)	IIB (15.4%)/IIC (15.4)/III (69.2%)	0.073

Of the 48 adult patients with deep high-grade STS included in this analysis, 46% were males and 54% were females. The mean age was 50.6 years (range 19-71). High-grade pleomorphic sarcoma was the most frequent histology, comprising 43.7% of the cases. Table 2 shows the relative frequency of specific histologic diagnoses.

**Table 2:** Relative frequency of histologic diagnosis

Histology	n (%)
		Pleomorphic Sarcoma	21 (43.7%)
Myxoid Liposarcoma	8 (16.7%)
Synovial Sarcoma	6 (12.5%)
Leiomyosarcoma	4 (8.3%)
High-grade Liposarcoma	3 (6.2%)
Malignant Peripheral Nerve Sheath Tumor	2 (4.2%)
Extraskeletal Myxoid Chondrosarcoma	2 (4.2%)
Rhabdomyosarcoma	1 (2.1%)
Extraskeletal Ewing's Sarcoma	1 (2.1%)
Total	48 (100%)

In most cases, the tumor involved the thigh (60.4%) followed by arm/shoulder/axilla (18.7%), forearm (8.3%), buttock (4.2%), leg (4.2%), scapular region (2.1%), and knee (2.1%). The mean size of the tumor, defined as the largest diameter, at the time of diagnosis was 12.3 cm (range: 4.5-29.1 cm).

Twenty-two patients were treated with pre-operative RT and then were operated on (group A) and 26 patients underwent initial surgery (group B). Eighty-one percent of patients in group B received RT post-operatively. Twelve of 22 patients (54.5%) in group A and 11 of 26 in group B (42.3%) also received chemotherapy as part of the treatment. Chemotherapy was not administered concomitantly with RT in any patient.

Mean follow-up time for the whole cohort was 49.4 months (minimum: four months due to death - maximum 114 months). No patient was lost to follow-up. Mean follow-up time was 44.3 months (range: 7-71, SD: 15.3) and 53.8 months (range: 4-114, SD: 36.5) in groups A and B, respectively. This difference in follow-up time between groups A and B is due to the change of treatment policy from post-operative RT to pre-operative RT.

Wound complications

In group A, 45.5% of the patients (n: 10) developed a wound complication of any grade. The rates of dehiscence, infection, wound necrosis and spontaneously draining seroma were 45.5%, 22.7%, 13.6% and 18.2%, respectively (Table 3). Six of these patients had minor complications that resolved with conservative measures in less than eight weeks. The remaining four patients developed major complications, all of which required at least one reoperation, one patient required prolonged antibiotic therapy, and one patient was unable to receive the planned chemotherapy (Table 4).

**Table 3:** Complication rate in patients in groups A and B according to the type of complication In group A, 10 patients who developed complications had more than one type of complication concurrently. Eight patients had two concurrent complications and two patients had three concurrent complications. In group B, nine patients developed one type of complication and five patients developed two concurrent complications.

	Group A		Group B		P-value
	n	%	n	%	
Any Complication	10	45.5	14	53.8	0.566
Wound Dehiscence	10	45.5	10	38.5	0.628
Infection	5	22.7	5	19.2	0.769
Skin Necrosis	3	13.6	1	3.8	0.223
Seroma	4	18.2	3	11.5	0.878
Major Complication	4	18.2	6	23.1	0.630

**Table 4:** Characteristics and outcome of the patients who developed major wound complications N/A = Not Applicable; VAC: Vacuum-assisted closure

Group	Gender/Age	Histology - Size	Site	Major Complication	Resolution	Adyuvant Treatment	Status (follow up)
A	F/53	Pleomorphic sarcoma (27 cm)	THIGH	Dehiscence/Infection	Toilette/Antibiotics (two surgeries)	Chemotherapy suspended	Dead (12 months)
	M/51	Myxoid liposarcoma (17 cm)	THIGH	Necrosis	Debridement/Skin Graft (two surgeries)	N/A	Alive (44 months)
	F/70	High grade liposarcoma (17cm)	THIGH	Dehiscence/Infection	Toilette/Antibiotics (one surgery)	N/A	Alive (43 months)
	F/60	Pleomorphic sarcoma - third recurrence (10 cm)	FOREARM	Dehiscence/Bone exposure	Muscular transference/Skin Graft (two surgeries)	N/A	Dead (46 months)
B	F/41	Extraskeletal Ewing´s sarcoma (14 cm)	KNEE	Dehiscence/Infection	Prolonged wound healing/Antibiotics	Chemotherapy suspended	Dead (14 months)
	M/31	Pleomorphic sarcoma (20 cm)	THIGH	Dehiscence/Bone exposure	Local muscular flap/Skin Graft (two surgeries)	Chemotherapy delayed	Dead (17 months)
	F/50	Myxoid liposarcoma (10 cm)	LEG	Necrosis	Debridement (one surgery)	Radiotherapy delayed	Dead (33 months)
	F/71	Pleomorphic sarcoma (25 cm)	THIGH	Dehiscence/Infection	Toilette/Antibiotics (two surgeries)	Radiotherapy suspended	Dead (9 months)
	F/48	Myxoid liposarcoma (12 cm)	THIGH	Dehiscence	VAC therapy/Skin Graft (four surgeries)	Radiotherapy suspended	Alive (52 months)
	M/59	Leiomyosarcoma (29 cm)	THIGH	Dehiscence/Bone exposure	Local muscular flap/Skin Graft (two surgeries)	Radiotherapy delayed	Dead (26 months)

In group B, 53.8% of the patients (n: 14) developed a wound complication of any grade. The rates of dehiscence, infection, wound necrosis and spontaneously draining seroma were 38.5%, 19.2%, 3.8% and 11.5%, respectively (Table 3). Eight of these patients developed minor complications. Major complications were seen in six patients. Five of them required at least one reoperation, three patients delayed the start of adjuvant treatment (chemotherapy: one patient, RT: two patients) and three patients did not receive the planned adjuvant treatment (chemotherapy: one patient, RT: two patients) (Table 4).

There were no statistically significant differences in the complication rate between groups A and B for complications of any grade (p=0.566) or for major complications (p=0.630). The mean tumor size in patients that developed major wound complications was 18.1 cm, which is considerably larger than the mean size of the whole series.

Completion of local treatment

**Figure 1:**
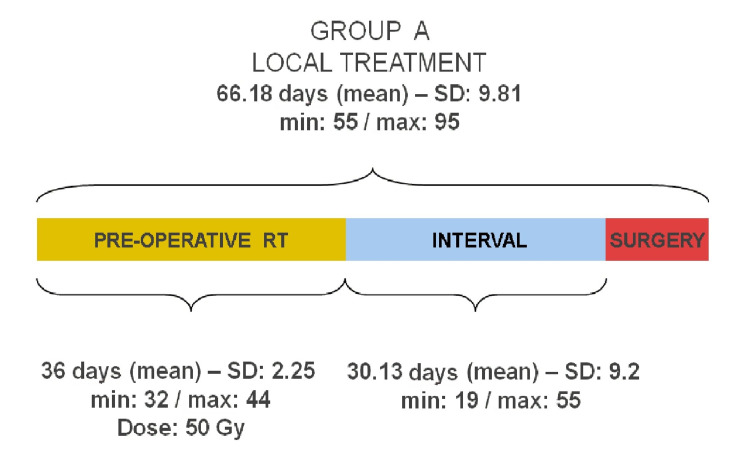
Treatment scheme and mean duration in patients treated with pre-operative RT (Group A) The commas within the values placed at the top of the bars represent decimals. RT - Radiation therapy

In group A, the mean duration of radiotherapy was 36 days (32-44, SD: 2.25 days) and the mean time between the end of RT and surgery was 30 days (19-55, SD: 9.2 days). All patients received the planned dose of RT and were operated on, completing their local treatment in less than eight weeks after the end of RT. The mean time elapsed between the start of RT and surgery in patients in group A was 66 days (55-95, SD: 9.81 days). This wide range is due to the fact that patients were operated on at the time that the skin was considered to be in its best condition (Figure 1).

In group B, two patients (7.7%) started radiotherapy more than eight weeks after surgery and two patients (7.7%) could not receive the planned RT because of wound complications. This results in 15.4% of patients not receiving the local treatment (surgery + RT) as planned (Table 4).

Oncological status

At last follow-up, nine patients (40.9%) in group A and 12 patients (46.1%) in group B had died. No statistical significant difference in overall survival was seen between patients in group A and B (p=0.335 Log Rank - Mantel-Cox).

Eight patients (16.7%) developed a local recurrence (four patients in group A and four patients in group B). No significant difference in the local recurrence rate was seen between the groups (p: 0.302). Of the eight locally recurring patients, three developed the local recurrence in the context of multiple distant metastatic dissemination. Excluding these three patients, the rate of local recurrence alone as a first event is 10.4%. Recurrences occurred between three and 24 months after surgery. Only one of the eight patients that recurred locally survived. The other seven patients died due to distant metastases between six and 22 months from the time of relapse.

Limb preservation was achieved in 95.8% of the patients. Two patients underwent a salvage amputation, one for a forearm recurrence and one had a palliative amputation for a recurrent lower limb sarcoma. No patient underwent amputation due to post-operative complications.

## Discussion

At present, the treatment of STS involves a limb conservation approach that consists of an oncologic surgery with negative margins, associated with RT according to the grade, size and depth of the tumor [2-4]. RT is also usually administered if a previous unplanned surgery was performed. A randomized study of limb-sparing surgery in high-grade STS showed that the addition of RT decreases the rate of local recurrence from 25% to 1.4%, confirming the need for RT if radical surgery is not to be performed [6,12].

In this study, we evaluated 48 consecutive adult patients who were seen by our multidisciplinary team with high-grade STS of the limbs. Demographic characteristics of the patients are in line with other reported series [5,6,13,14].

In the beginning, patients with localized high-grade STS were treated with a post-operative RT regimen when deemed necessary. But, given the potential advantages of administering the RT before surgery, after 2013, most patients were treated with pre-operative RT [7,10,11]. Twenty-two patients received RT before surgery. These patients were compared with 26 patients that had not received RT before surgery in order to analyze the acute wound complication and the local treatment completion rates. Both groups of patients were similar in all the variables studied.

Post-operative RT has the theoretical advantage of removing the tumor earlier and potentially decreasing the chance of metastatic seeding, although this has not been demonstrated [15]. In addition, pathologic evaluation of the surgical specimen is more accurate if no neo-adjuvant treatment is delivered before surgery. Nevertheless, nowadays core-biopsies are the standard of care [16]. On the other hand, radiotherapy is more precise when it is delivered before surgery because the treatment volume is directed to the tumor itself and not to the surgical bed, which is sometimes difficult to delimit in the RT planning CT scan. As a result, the treated volume is smaller. In addition, in the post-operative setting, tissues are less oxygenated, resulting in the need for higher radiation doses. A standard regimen of post-operative RT consists of 60-66 Gy compared to the 50 Gy usually delivered before surgery [8]. This difference in RT volume and dose is responsible for the higher chronic complication rate observed with post-operative RT. Reported late complications include tissue necrosis, fractures, neurologic damage, edema, fibrosis, joint stiffness and secondary tumors [9,17].

Pre-operative RT has also a potential oncologic advantage described by Dagan et al. in a study in which the administration of RT before surgery yields the same local control rates when negative margins are achieved, as when a “marginal” margin is achieved at some point [18]. On the contrary, a marginal margin is known to be a negative prognostic factor for local control when RT is delivered postoperatively. Nevertheless, obtaining a marginal margin is not pursued, but in certain circumstances might be the only option in order to preserve the limb or its function.

To our knowledge, the only reported drawback of pre-operative RT is an increased rate of acute wound complications. This has discouraged many surgeons from considering the use of RT before surgery. In our series of 48 patients, we have not seen a difference in the incidence of acute wound complications (any grade or major complications) between the two groups of patients. The most important study that reported on this topic is the NCI-C trial, first reported by O´Sullivan in 2002 [8,9]. This study randomized 190 patients to pre-operative or post-operative RT and analyzed the rate of acute postoperative wound complications. Results were compelling, with a double rate of acute complications if RT was delivered before the surgery (35% versus 17%). The definition of major wound complication used in that study included patients that required aspiration of a seroma. We believe this is not a serious complication that might delay the future treatment of the patient. In this study, we defined as major complications only those that were severe enough to delay the delivery of adjuvant treatment. Other studies also report more acute complications with the use of pre-operative RT [13,17,19-21]. However, in accordance with our experience, Le Brun et al. did not observe a significant difference in the rate of acute wound complications with pre-operative or post-operative RT [22]. A possible explanation for not seeing more wound complications with pre-operative RT in our patients may be the fact that they were all treated by the same team, what facilitates the supervision of the patients, the precise selection of the time of surgery according to the condition of the skin, the utmost care of the soft tissues not involved in the resection, and meticulous surgical wound closure, avoiding areas of tension. In addition, during RT planning, special attention was paid to administer the dose as homogeneously as possible, avoiding “hot spots” near the skin. A study from the University of North Carolina reported a significantly lower re-operation rate in patients that had received pre-operative RT in the same institution where the surgery was performed, as compared to patients who were irradiated and operated in different institutions (11% vs. 33%, p:0.029) [23]. This would support the importance of the close collaboration between surgeons and radiation oncologists. In line with the literature, 90% of the major wound complications in our patients occurred in the lower limb [8].

In the present study, 15% of the patients that were operated on first did not complete the oncologic local treatment as planned since they were unable to receive the post-operative RT due to wound complications. The treatment of these complications resulted in the delay or lack of RT administration. In contrast, 100% of the patients that received pre-operative RT underwent surgery within the estimated time and completed the local treatment in a mean time of 66 days. Casabianca et al. reported the experience of a tertiary center dedicated to the treatment of sarcomas. In this series, 26% of the patients did not start RT in the first 12 weeks after surgery. Most importantly, 8% never received the planned postoperative RT [24]. In a similar study, Miller et al. showed that 15% of the patients delayed the start of RT for more than three months after surgery and one patient (1.4%) did not receive RT due to major wound complications [25]. Other studies report delays of three to four months to start post-operative RT in 7%-16% of the patients [11,26,27]. Cannon et al. reported that patients that developed wound complications started the post-operative RT 60 days after surgery compared to 29 days in the patients that did not develop any complication [17]. We believe that the completion of the local treatment of STS is of utmost importance. This is the reason why RT should preferably be administered before surgery in patients in whom RT is deemed necessary. As a matter of fact, if a wound complication is to happen, the oncologic local treatment completion is not jeopardized since the event occurs after both treatments (surgery plus RT) have been performed. In this way, wound complications are limited to be orthopedic problems and not oncological problems due to incomplete local treatments.

At study closure, the limb conservation rate was 95%. In 1982, Rosenberg et al. demonstrated in a randomized trial comparing amputation versus limb-sparing surgery plus RT that despite a larger number of local recurrences (0% vs. 11%) the overall survival is not compromised in the conservative approach [5].

Certain factors like diabetes, obesity, smoking and peripheral vascular disease have been associated with the development of wound complications [28,29]. These factors were not analyzed in our patients. Other limitations of this study include its non-randomized nature, the relatively small number of patients, and that follow-up is not long enough to analyze the long-term toxicity. Despite the small number of patients, the study population was well defined and resulted statistically comparable. However, as in any non-randomized study biases cannot be excluded.

## Conclusions

In this study, we did not observe an increased risk of acute wound complications with the administration of pre-operative RT in patients with STS of the limbs. As opposed to the patients who received RT after the surgery, all the pre-operative RT patients completed the planned combined local treatment in the stipulated time.

The development of a wound complication after the completeness of local treatment delivery does not jeopardize the rate of local control and is limited to be a transitory orthopedic problem. We believe pre-operative RT should be the standard treatment option for STS.
